# Academic performance and its associated factors among khat-chewer and non-khat chewer high school students in Ethiopia, 2023

**DOI:** 10.3389/fpsyt.2025.1452018

**Published:** 2025-04-10

**Authors:** Tilahun Tesfa, Tilahun Belete Mossie, Meseret Tadesse, Yonas Deressa Guracho, Birhanu Mengist Munie

**Affiliations:** ^1^ Department of Psychiatry, Motta General Hospital, Amhara Regional Health Bureau, Motta, Ethiopia; ^2^ Department of Psychiatry, College of Medicine and Health Sciences, Bahir Dar University, Bahir Dar, Ethiopia; ^3^ Faculty of Engineering and Information Sciences, University of Wollongong, Wollongong, NSW, Australia; ^4^ Department of Psychiatry, College of Health Sciences, Debre Tabor University, Debre Tabor, Ethiopia

**Keywords:** academic, academic performance, khat chewing, student, Ethiopia

## Abstract

**Background:**

Academic performance measures student achievement and is assessed using school exams and ongoing evaluations. Regular khat consumption can negatively affect neurocognitive function, decision-making, and problem-solving abilities, leading to decreased academic achievement, especially in Ethiopia. This study aimed to assess academic performance and associated factors among khat chewer and non-khat chewer high school students in Motta in northwestern Ethiopia in 2023.

**Methods:**

A comparative cross-sectional study was conducted between April 26, and June 10, 2023, among 663 high school students, of whom 221 were khat chewers and 442 were non-chewers. A multistage sampling method was used to select participants. A structured, self-administered questionnaire was used to assess students’ academic performance. Data were analyzed using IBM SPSS version 25. Ordinal logistic regression analysis was performed at P < 0.05 with a 95% confidence interval to identify statistically significant variables.

**Results:**

The study participants, 45 (20.7%) Khat chewers and 41 (9.4%) non-chewers, had failed/poor academic performance. Of the students who scored less than 50% on one subject, 83 were (38.2%) khat chewers and 107 (24.6%) were non-chewers. The mean of academic performance was 59.82 and 66.45 for khat chewers and non-chewers, respectively. The findings showed that the chance of having an excellent academic performance was 35% lower for female students [AOR=0.65:95%CI: -0.819–0.055] and 45% lower for those unsatisfied with their school environment [AOR: 0.55, 95% CI: -1.014–0.173]. The chance of having an excellent academic performance was 6.43 times higher for those whose fathers had an educational grade of 7–12 [AOR: 6.43, 95%CI: 1.098–2.624] and 6.59 times higher for those with fathers who had a diploma [AOR: 6.59, 95% CI: 1.026–2.745], 8.01 times higher for those with a family income of 2001–5000ETB [AOR=8.01:95% CI: 1.479–2.683], 2.07 times higher for those with no alcohol use disorder [AOR: 2.07, 95% CI: 1.21–2.93], and 6.84 times higher for those with no depressive disorders [AOR: 6.84, 95% CI: 1.056–2.791].

**Conclusions:**

Academic performance was lower among khat chewers than among non-khat chewers, and was affected by factors such as family income, father’s educational status, classroom environment, depression, anxiety, and alcohol use disorder. Enhancing school-community relations and increasing awareness can enhance academic performance.

## Introduction

Academic performance refers to a student’s progress toward degree completion, irrespective of circumstances or issues related to the institution (1). Good academic performance is a key driver of economic, social, and political development. Governments, communities, and missionary organizations have been increasingly dedicated to education (2). This commitment has been extended to ensure that students are properly educated on how best to manage themselves in an ever-changing world. These changes have led some students to believe that they need to take harmful substance to maintain their academic excellence (3). According to the above descriptions, one can determine that a student’s academic achievement includes a variety of factors based on their grades, skills, and emotional intelligence demonstrated to receive a given qualification (4).

According to most studies, excessive and prolonged khat use results in neurocognitive deficit syndrome, marked deterioration of psychophysical functions, negative effects on perception, problem-solving, cognitive control, working memory monitoring/updating, flexibility and inhibition, and decision-making ability, and poor academic performance (5). The availability of khat and its introduction to young individuals, along with the perceived benefits linked to its consumption, encourage youths to develop a khat-chewing habit, leading to various health issues (6).

Substance abuse, including khat abuse, is currently one of Ethiopia’s most pressing public health concerns, with students in higher education institutions being the most significantly impacted (7). According to several studies, khat chewing helps students achieve a sense of well-being, euphoria, increased alertness and energy, emotions of pleasure, increased communication, improved intellectual ability and ability to associate ideas, and increased self-confidence (8).

Globally, different studies have focused on high schools and colleges/universities; however, students in different parts of the country have also reported varying prevalence levels of khat chewing and associated factors, in which ranges of 20% to30% have been reported (9). A study of the relationship between secondary school and college students’ khat-abuse patterns and academic achievement was conducted. In the Kingdom of Saudi Arabia, 37.0% of people chewed khat with their friends at home, with the average khat session lasting 3.13 to 6.1 hours. Of those participants, 48.4% chewed khat on the weekends while 69.8% did so after noon. Of these khat chewers, 39.4%, 41%, and 39.60% of the 78% khat chewers had low grades, were absent from class, or were unsuccessful because of their poor grades, respectively (10).

According to a study conducted in three communities in southwestern Uganda, students were most likely to chew khat, with a prevalence of 9.2% (11). In Ethiopia, studies conducted among students in different universities/colleges have reported lifetime prevalence values ranging from 13.4% to 41%. Similarly, 12-month prevalence values ranging from 7% to 20.3% have been reported and the previous 30-day prevalence has been reported in the range of 6.3%–33.1% (12).

The lifetime prevalence of khat chewing among Jimma high school students was around 16%. The current khat-chewing prevalence among these students is 14.2%, of which many students had mental distress. Of them, 22.2% are khat chewers aged 19–23 years (13). The study was conducted on Jijga University students’ khat-chewing prevalence Of the students, 33.3% had consumed khat at least once28.33% were currently chewing khat, and about half of them (50.5%) first started khat chewing in high school (14).

The survey was conducted on high and preparatory school students in Ginner Town, Bale Zone, and southeast Ethiopia; 64% of male participants and 13% of female participants had friends who used khat (15). Khat use significantly impacts students’ characteristics, according to research conducted on higher education students at Mizan Tipi University. Although using khat seems to help students to stay alert and concentrate during extended study sessions, the overall findings of this study indicate that khat use negatively impacts students and their academic achievements (16).

Despite the prevalence of khat chewing among high school students, many adolescents lack awareness of its harmful consequences (17). Khat chewers had higher failed academic performance than non-khat chewers among Hawassa University students: 55% failed and 45% had good academic performance. Of non-khat users, 33.3% failed and 66.7% performed well academically (18).

Among Ethiopian students and adolescents, chronic khat use leads to gastrointestinal tract problems, cytotoxic effects on the liver and kidneys, keratotic lesions at the chewing sites, malnutrition, anorexia, constipation, and stomatitis. It also increases vulnerability to infectious diseases, particularly tuberculosis, which can result in death after prolonged use. In addition to these negative health effects, they also adversely affect academic performance (19).

According to several studies, chewing khat has dangerous psychological effects on both individuals and communities. Depressive and psychotic reactions are some of the negative effects of khat use that indirectly affect academic performance (20). Numerous studies have shown that regular khat use negatively impacts a user’s social and economic situation; the ability to afford healthy food, housing improvements, education, and other needs may be impacted by the daily cost of khat use, which ultimately results in financial difficulty and family disintegration, consequently leading to poor academic performance (21).

Chewing and purchasing khat leaves take up a significant amount of time, affecting working hours and time, which results in job loss, poor academic achievement, and absenteeism from work and class (22). However, few studies have examined the relationship between academic performance and khat chewing among high school students in Ethiopia.

Factors contributing to students’ substance use have been identified and propagated, including easy access to substances within direct neighborhoods, failure of school achievement, feeling dull, and unwanted peer influence (23). Many factors affect students’ academic performance such as their current mental health status, working status, poor time management, duration of the study time, and time spent on social media per day. Statistically, about 41.2% and 44.3% of students claim that their academic performance is affected by their emotions and mental health, respectively (24). The school environment is one of the problems that attracts students to frequent absenteeism, consequently leading them to drop out. Late arrival is also a characteristic of poor academic performance (25).

Several studies have been conducted on the prevalence of khat chewing in different populations in Ethiopia, including college and high school students. However, few studies have been conducted on the relationship between academic performance and khat chewing among high school students. This study also intended to compare khat chewers with non-khat chewers. Therefore, this study aimed to explore possible interventions to control and prevent khat chewing among students. It also provides baseline information for stakeholders and policymakers and serves as a resource for scholars in relevant fields. Additionally, it is useful for active participation based on the recommended areas.

## Materials and methods

### Participants and study period

The three high schools in the town have 5059 students, ranging from Grades 9–12 (2581 women and 2478 men) (26). All students in the three selected high schools in Motta who were present during the study period were selected for the study. The study was conducted from April 26 to June 10, 2023. An institution-based comparative cross-sectional study was conducted to compare the academic performance of students who were khat and non-khat chewers.

### Source and study population

Students who attended high schools in Motta and registered for a regular program were included in the study. All evening high school students were excluded from the study.

### Sample size

The sample size was calculated by using double-population proportion for an unequal sample size formula and Epi Info 7 software, using the reference of Hawassa University, poor academic performance of khat and non-khat chewers n_1_= (khat chewers) =55%, n_2_= (non-chewer) = 33.3%, CI= 95%, Power = 80%, and ratio 1:2 (18).

Za=1.96, p=p_1_+p _2_/2 = 0.44, q=1-p=0.56, Zb (power) =0.84, p_1_ = 0.55, q_1_ = 1-p_1=_0.45, p_2_ = 0.33, q_2_ = 1- p_2=_0.67.

Double-population proportion for the unequal sample formula was used to compare the academic performance of khat and non-khat chewers among high school students.


$$\text{n}1=\frac{[\text{za}/2\sqrt{\text{pq}\left(1\;+\;1/ለ\right)}\;+\text{zb}\sqrt{\text{p}1\text{q}1+\text{p}2\text{q}2/ለ{]}^{2}}}{{\left(\text{p}1-\text{p}2\right)}^{2}}$$

n1 =  46 and n2 =  92, then times three for design effect n_1_ = 138 and n_2_ = 276, and adding 10% for nonresponse rate; the total sample size was =456.

However, the sample size was also calculated using Epi Info 7 and n_1_ = 67 and n_2_ = 134, then multiplied by three for design effect n_1_ = 201 and n_2_ = 402, then 10% added for nonresponse rate for each sample; the sample size becomes 221 khat-chewers and 442 non-khat chewers. The final sample size was 663.

### Sampling procedures

A multistage sampling technique was used to select the study subjects. Pre-sampling screening was conducted in three selected high schools, followed by the development of two independent sampling frames based on the status of students as khat users (n = 476) and non-chewers (4583). The sample size was then distributed proportionately considering the relative size and grade level of the students in each school. This ensured that khat chewers and non-chewers were proportionately distributed across high schools. Ayer Marefiya High School had 1051 (non-chewers = 95, khat chewers = 43) and Motta Preparatory School had 1900 (non-chewers =152, chewers = 99).

**Figure 1:**
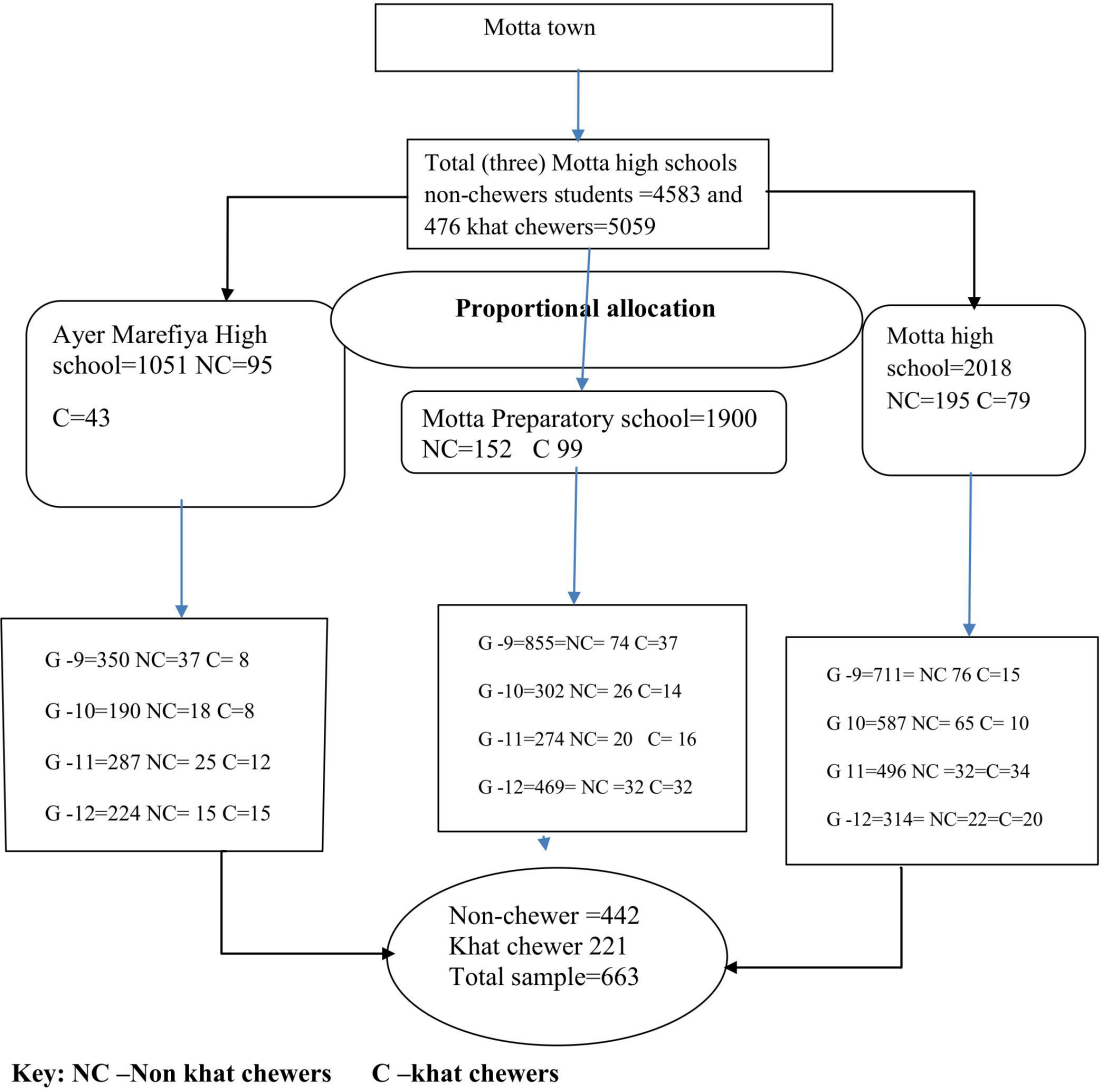
Schematic presentation of sampling procedure for the study academic performance and associated factors among khat chewers and non-khat chewer high school students at Motta town north-west Ethiopia in 2023.

The distribution in Motta High School was 2108 (non-chewers =195, chewers =79); N=5059. A simple random sampling method was used to create a list of students’ use as the sampling frame in each group, and the k-value was calculated as n_1_= N/n (476/221 = 2) and n_2_= (4583/442 = 10). Khat chewers were selected every two intervals and non-khat chewers every 10 intervals (**Figure 1**).

#### Variables

The dependent variable.is academic performance and the independent variables are sociodemographic factors (e.g., age, sex, religion, current residence, educational level) and substance-related factors (e.g., alcohol and khat use). Family-related factors are the educational background of parents, occupation, and monthly income. Environmental and psychological factors are school environment, depression, and anxiety.

#### Data-collection tools and procedures

Data were collected using a structured, self-administered questionnaire, and the academic performance of the students was based on the first-semester average score according to the Ministry of Education’s scoring system. Scores of 90%–100% were considered excellent, 80%–89% very good, 60%–79% satisfactory, 50%–59% fair, and below 50% were poor/failed academic performance levels of students. Their academic grades were collected from the student roster.

The Patient Health Questionnaire (PHQ9) was used to assess depression. It uses the nine items of the PHQ-9 tool, cross-culturally validating the tools in Ethiopia with a sensitivity and specificity of 86% and 67%, respectively (27). A PHQ-9 score ≥ 5 is considered as having depression.

General Anxiety Disorder-7 (GAD-7) determines the level of anxiety. The scores of all items were added to give a final score ranging from 0 to 21. A recent study on university students reported the internal consistency of GAD-7 as measured by Cronbach’s alpha (0.80) (28).

The Alcohol Use Disorder Identification Test (AUDIT), developed by the World Health Organization, was used to screen for alcohol use. The questionnaires were validated. In southern Ethiopia, the Dilla University Hospital AUDIT checked the sensitivity and specificity, which were 94% and 84%, respectively, and had high internal consistency reliability (Cronbach’s α = 0.98) (29).

Additionally, school environment questionnaires were adopted from different studies to assess environmental factors. The questionnaires were developed in English, translated into Amharic, and independently translated back into English to ensure consistency. For the selected khat and non-khat chewers, the data were completed by the students after they were given a detailed orientation by five psychiatric professional coordinators and three school-unit leaders.

### Data quality control

Prior to data collection, five professional psychiatry coordinators and three school-unit leaders underwent a one-day training session on the study tool and data-collection procedure. A pretest was carried out at Hulet Ejju Enessie Woreda Merit High School with selected students, representing 5% of the sample size, to evaluate the questionnaire’s quality, the coordinators, and the respondents. This was performed to determine the effectiveness of the data-collection tools in obtaining the necessary information.

#### Data processing and analysis

After data collection, data were edited, cleaned, and coded before being input into SPSS-25 for analysis. The study findings are presented using a descriptive analysis, which includes frequencies, graphs, and cross-tabs. Bivariate and multivariate ordinal logistic regression analyses were used to determine the factors and their relationships with the dependent variable, and odds ratios (ORs) were calculated to assess the strength of the association and statistical significance. Variables with a P-value below 0.25 in the bivariate analysis were included in the multivariate analysis, while a p-value less than 0.05 was considered statistically significant.

##### Operational definitions

The following operational definitions are needed for clarification of some content.

Academic performance: Students’ academic levels are illustrated as follows: 90%–100% (excellent), 80%–89% (very good), 60%–79% (satisfactory), and 50%–59% (fair), and below 50% (poor/failed) (30).

Current khat chewer: A student who chews khat one or more times within three months (17).

Alcohol use disorder: Students’ scores of > 8 suggest the possibility of alcohol use disorder (29).

Depression: Students who scored ≥ 10 were considered as having depression (27).

Anxiety: Students who scored ≥ 10 were considered to have anxiety disorders (28).

School environment: Students below a mean of 30 had unsatisfactory results and those above a mean of 30 experienced a satisfactory school environment (31).

## Results

### Sociodemographic characteristics of study participants

Of 221 khat chewers and 442 non-khat chewers, the study participants had a response rate of 98.2% and 98.6%, respectively. The mean age of the students was 20.21 with a standard deviation of 3.69 for khat chewers and 19.29 with a standard deviation of 3.05 for non-chewers. The majority of participants among khat chewers were men, accounting for 144 (66.4%), while 73 (33.6%) were women. Among non-chewers, 191 (43.8%) participants were men and 239 (56.2%) were women. Among the khat chewers, 172 (79.3%) resided in urban areas and 45 (20.7%) in rural areas (**Table 1**).

**Table 1:** Sociodemographic characteristics of student among khat chewers vs. non-chewers of high school students in Motta in northwestern Ethiopia, 2023.

Variable	Category	Khat chewers		Non-khat chewers	
		Frequency	%	Frequency	%
Age	14–19	109	50.2	238	54.6
	‗20–24	76	35.0	164	37.6
	‗24	32	14.7	34	7.8
Sex	Men	144	66.4	191	43.8
	Women	73	33.6	245	56.2
Grade	Grade 9	58	26.7	185	42.4
	Grade 10	32	14.7	108	24.8
	Grade 11	60	27.7	75	17.2
	Grade 12	45	20.7	68	15.6
Current residence	Rural	45	20.7	107	24.5
	Urban	172	79.3	329	75.5
Religious	Muslim	94	43.3	70	16
	Orthodox	122	56.2	356	81.7
	Protestant	1	0.5	10	2.3
Mother educational status	Cannot read and write	106	48.8	216	49.5
	Grades 1–6	79	36.4	112	25.7
	Grades 7–12	13	6.0	44	10.1
	Diploma	6	2.8	28	6.4
	Degree and above	13	6.0	36	8.3
Father educational status	Cannot read and write	53	24.4	155	35.6
	Grades 1–6	96	44.2	141	32.3
	Grades 7–12	30	13.8	59	13.5
	Diploma	20	9.2	34	7.8
	Degree and above	18	8.3	47	10.8
Family monthly income	<2000 ETB	64	29.5	143	32.8
	2001–5000 ETB	84	38.7	198	45.4
	>5001 ETB	69	31.8	95	21.8
	>5001 ETB	69	31.8	95	21.8

### Academic performance among khat-chewer and non-khat chewer high school students

Academic performance of khat chewers was lower than that of non-chewers. Among the current khat chewers, 20.7% (45/217) had a failed/poor academic performance, whereas of non-chewers, 9.4% (41/436) scored a failed/poor academic performance. Of the participated 217 khat chewers, 38.2% (83/217) scored less than 50% for one subject, whereas 24.6% (107/436) of non-chewers scored less than 50% for one subject, which is lower by 13.6% than that of khat chewers (**Table 2**). The mean academic performance of khat chewers was 59.82 with a standard deviation of 12.96 and of non-chewers was 66.45 with a standard deviation of 13.06.

**Table 2:** Academic performance among khat-chewer and non-chewer high school students in Motta northwestern Ethiopia, 2023.

Variables	Category	Non-khat chewers		Khat chewers	
		Frequency	%	Frequency	%
Academic performance	Excellent	27	6.2	9	4.1
	very good	46	10.6	19	8.8
	Satisfactory	188	43.1	76	35.0
	Fair	134	30.7	68	31.3
	failed/poor	41	9.4	45	20.7
Below 50% by one subject	Mathematics	43	9.9	34	15.7
	English	18	4.1	14	6.5
	Physics	12	2.8	8	3.7
	Chemistry	12	2.8	7	3.2
	Other social sciences	22	5.0	20	9.2
	Total	107	24.6	83	38.2

### School environment among khat-chewer and non-khat chewer high school students

The mean of school environment for academic performance among high school students was 38.77, with a standard deviation of 9.69. The minimum and maximum mean values were 10 and 50, respectively. By summing the minimum and maximum means and dividing by two, the reference score becomes 30. Therefore, a mean above 30 for school environment was considered satisfactory and below 30 was considered unsatisfactory.

**Figure 2:**
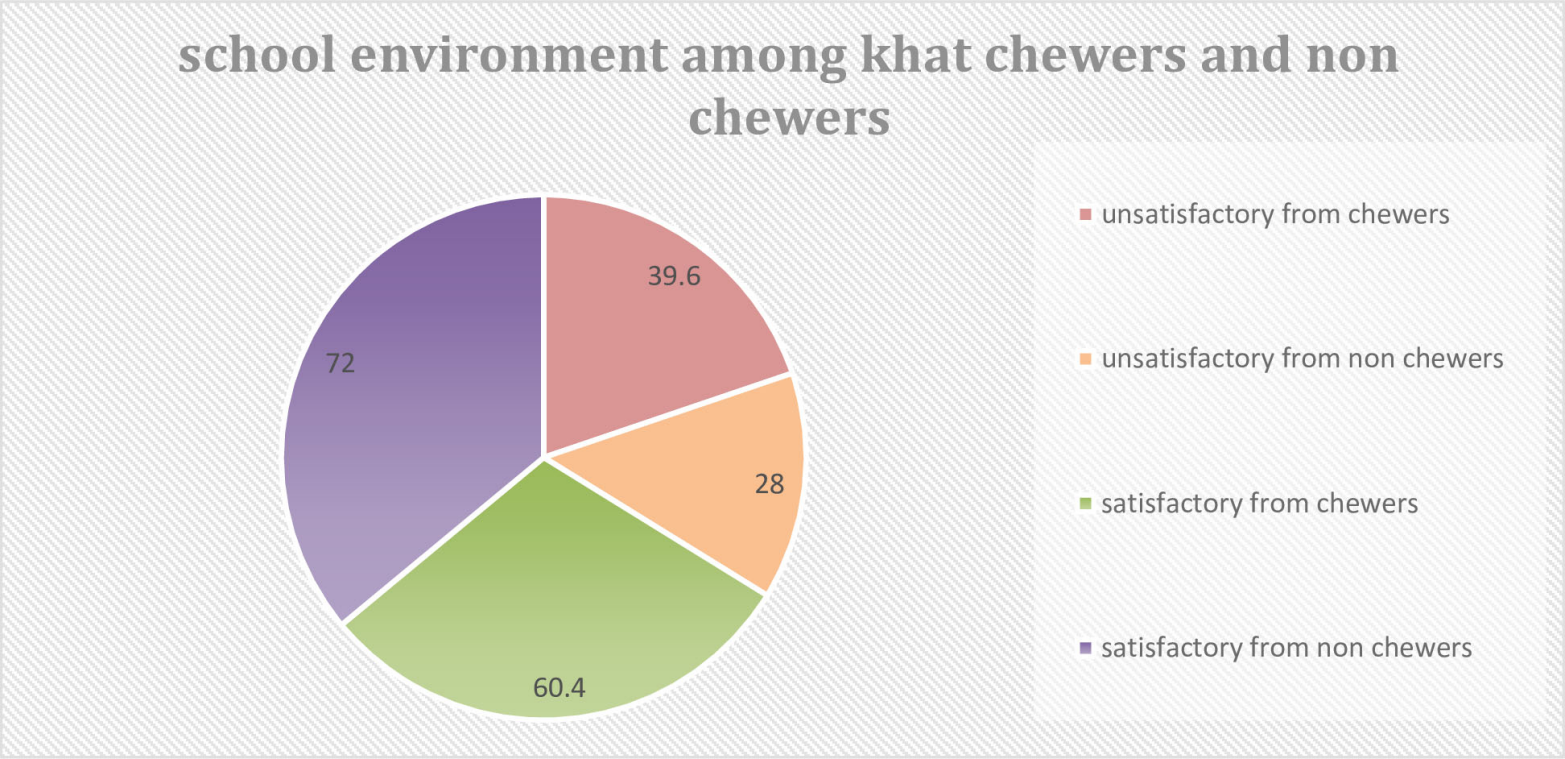
School environment among khat chewer and non-chewer high school students at Motta town, North West Ethiopia, 2023.

Based on the findings, of the khat chewers, 131 (60.4%) students were satisfied with the school environment and 86 (39.6%) were unsatisfied; of the non-chewers, 314 (72%) students were satisfied with the school environment and the remaining 122 (28%) were unsatisfied regarding the school environment. Of these, 30 (34.9%) khat chewers and 24 (19.67%) non-khat chewers had failed/poor academic performances (**Figure 2**).

### Substance and psychological related characteristics among khat-chewer and non-khat chewer high school students

The prevalence of alcohol use disorder was 20.7% (45/217) among khat-chewer students. In contrast, 16.3% (45/436) of non-khat chewer students presented a high level of alcohol use disorder. The prevalence of general anxiety disorders among khat-chewer respondents was 16.1% (35/217). Among non-chewers, the prevalence of general anxiety disorders was 10.3% (45/436). The magnitude of depressive disorder among Motta High School students from khat chewers was 17.5% (38/217). Among non-chewers, 11.2% (49/436) of students had depressive symptoms. **Table 3** presents the substance and psychological characteristics of khat chewers and non-khat chewers in Motta in northwestern Ethiopia in 2023.

**Table 3:** Substance and psychological related characteristics among khat-chewer and non-khat chewer high school students in Motta in northwestern, 2023.

Variables	Category	Non-chewers		Khat chewers	
		Frequency	%	Frequency	%
Alcohol use disorder	No	365	83.7	172	79.3
	Yes	71	16.3	45	20.7
General anxiety disorder	Yes	45	10.3	35	16.1
	No	391	89.7	182	83.9
Depressive disorder	Yes	49	11.2	38	17.5
	No	387	88.8	179	82.5

### Factors associated with academic performance among non-khat chewers

Any variable whose bivariate test has a p-value ≤ 0.25 was a candidate for multivariate ordinal logistic regression analysis. Once the variables were identified using the univariate ordinal logistic regression analysis, we used a model containing all the selected variables for the multivariate ordinal logistic regression analysis.

According to the results of this analysis, variables such as paternal educational status, family monthly income, school environment, depressive disorder, and alcohol use disorder were significantly associated with academic performance among non-chewers at a 5% significance level. The findings showed that female students were 35% less likely to achieve excellent academic performance than male students [AOR=0.65:95%CI: -0.82, 3.05]. The chance of having excellent academic performance among students with a family income of 2001–5000ETB was eight times (8.01) higher than that of students whose family income was below 2000ETB per month [AOR=8.01:95% CI:1.48, 8.68].

The chances of having an excellent academic performance for students whose fathers had an educational level of Grades 7–12 or a diploma were more than six times [AOR: 6.43, 95%CI: 1.12, 7.62] and above six times [AOR: 6.59, 95%CI: 1.03, 6.75] higher, respectively than those whose fathers cannot read and write at educational levels. Moreover, students who were unsatisfied with their school environment scored 45% lower [AOR: 0.55, 95% CI: -1.02, 1.17] in academic performance than those who were satisfied with their school environment. Students who had alcohol use disorders were 79% less likely to have an excellent academic achievement than those who had never used alcohol [AOR: 0.21, 95% CI: 2.09, 0.99]. Similarly, students who had no depressive disorders were nearly seven times (6.84) more likely to perform on an excellent level academically than were those who had never had a depressive disorder [AOR: 6.84, 95% CI: 1.06, 7.79]. **Table 4** shows the factors that are associated with academic performance by bivariate and multivariate ordinal logistic regression among non-khat chewer students in Motta in northwestern Ethiopia in 2023.

**Table 4:** Factors associated with academic performance by bivariate and multivariate ordinal logistic regression among non-khat chewer students in Motta in northwestern Ethiopia, 2023.

Variables	Category	Estimate	AOR	P-value
Sex	Men	Ref.		
	Women	-0.437	0.65 (-0.82, 3.05)	0.025
Age	14–19yrs	Ref.		
	20–24yrs	0.767	2.15 (0.047, 1.487)	0.037
	>24yrs	0.511	1.67 (-0.223, 1.245)	0.172
Residence	Rural	Ref.		
	Urban	-0.381	0.683 (-0.824, 2.63)	0.093
Father educational status	Cannot read and write	Ref.		
	Grades 1–6	1.552	4.72 (0.768, 4.336)	<0.001
	Grades 7–12	1.861	6.43 (1.12, 7.62)	<0.001
	Diploma	1.886	6.59 (1.03,6.75)	<0.001
	Degree and above	1.228	3.42 (0.291, 4.166)	0.010
Mother educational status	Cannot read and write	Ref		
	Grades 1–6	0.958	2.61 (0.104, 2.812)	0.028
	Grades 7–12	0.795	2.22 (-0.079, 2.668)	0.075
	Diploma	0.707	2.03 (-0.258, 1.672)	0.151
	Degree and above	0.237	1.27 (-0.84, 1.313)	0.667
Family monthly income	<2000 ETB	Ref		
	2001–5000 ETB	2.081	8.01 (1.48, 8.68)	0.001
	>5001 ETB	0.537	1.71 (0.024, 2.05)	0.040
School environment	Satisfied	Ref		
	Unsatisfied	-0.594	0.21 (-1.02, 1.17)	0.006
Anxiety	Yes	Ref.		
	No	0.647	1.91 (-0.23, 2.52)	0.148
Depression	Yes	Ref		
	No	1.923	6.84 (1.056,7.79)	<0.001
Alcohol use disorder	No	Ref		
	Yes	-1.54	0.21 (-2.09, 0.99)	0.001

### Factors associated with academic performance among khat chewers

**Table 5** shows the factors of academic performance by bivariate and multivariate logistic regression among khat-chewer students in Motta in northwestern Ethiopia in 2023. Any variable whose bivariate test has a p-value ≤ 0.25 was a candidate for multivariate ordinal logistic regression analysis. Once the variables were identified by bivariate ordinal logistic regression analysis, we used a model containing all selected variables for multivariate ordinal logistic regression analysis to decrease the confounding effects of the variable. According to the results of this analysis, variables such as sex, school environment, anxiety, depression, and alcohol use disorder were significantly related to academic performance among khat chewers at a 5% significance level.

**Table 5:** Factors associated with academic performance by bivariate and multivariate ordinal logistic regression among khat-chewer students in Motta in northwestern Ethiopia, 2023.

Variables	Category	Estimate	AOR	P-value
Sex	Men (Ref)			
	Women	-0.66	0.52 (-1.27, 1.06)	0.03
Age	14–19	Ref	Ref	Ref
	20–24	0.203	1.22 (-0.64, 1.043)	0.65
	>24	-0.56	0.58 (-1.44, -0.329)	0.22
Residence	Rural	Ref		
	Urban	-0.19	0.61 (0.18, 1.94)	0.56
Father educational status	Cannot read and write	Ref	Ref	Ref
	Primary education	1.10	3.01 (-0.15, 4.36)	0.15
	Secondary education	0.32	1.37 (-0.82, 2.45)	0.59
	Diploma	0.24	1.28 (-1.03, 1.52)	0.71
	Degree and above	0.24	1.27 (-1.04, 1.53)	0.71
Mother educational status	Cannot read and write	Ref	Ref	Ref
	Primary education	-0.53	0.59 (-1.90, 0.85)	0.45
	Secondary education	0.58	1.79 (-0.74, 1.89)	0.39
	Diploma	0.13	0.15 (-1.46,1.68)	0.89
	Degree and above	-1.62	0.20 (-3.51,0.28)	0.09
Family monthly income	<2000ETB	Ref	Ref	Ref
	2001–5000 ETB	0.05	1.05 (-0.87,0.96)	0.92
	>5000ETB	-0.13	0.88 (-0.86, 0.61)	0.74
School environment	Unsatisfactory	-1.02	0.36 (-1.61, -0.43)	0.005
	Satisfactory	Ref	Ref	Ref
Anxiety	Yes	-1.19	0.30 (-2.22,-0.17)	0.022
	No	Ref	Ref	Ref
Depression	Yes	-1.44	0.24 (-2.39, 1.48)	0.003
	No	Ref	Ref	Ref
Alcohol use	Yes	2.07	7.94 (1.21,8.93)	0.001
	No	Ref	Ref	Ref
Current khat use	Yes	1.81	6.11 (1.25,8.37)	<0.001
	No	Ref	Ref	Ref

The findings showed that the chance of having excellent academic performance for female students was 48% higher compared to male students [AOR=0.52, 95%CI: -1.27, 1.06]. Students who had no alcohol use disorder were 7.94 times more likely to achieve excellent academic performance than those who had an alcohol use disorder [AOR: 7.94, 95%CI: 1.21, 8.93]. Students who were non-khat chewers were 6.12 times more likely to achieve excellent academic performance than those who were current khat chewers [AOR: 6.15, 95%CI: 1.25, 8.93]. Similarly, those who had anxiety disorders were 70% less likely to perform well academically than those who had no anxiety disorders [AOR: 0.30, 95% CI: -2.22, 1.17]. Students who had depression were 76% less likely to perform well academically than those who had never had depressive symptoms [AOR: 0.24, 95% CI: -2.39, 1.48]. Moreover, students whose school environment was unsatisfactory were 64% less likely to succeed in academic performance than those who were satisfied with the school environment [AOR: 0.36, 95% CI: -1.61, 1.43].

## Discussion

This study mainly aimed to examine the determinants of academic performance among high school students at three selected high schools, using an ordered logit model. The study assumed the ordered nature of the categories of academic semester results as excellent, very good, satisfactory, fair, and failed, based on the Ministry of Education’s standard cutoff point. The results of this study compared the academic performance of khat and non-khat chewers among Motta High School students.

Based on the questionnaire’s findings, the majority of the respondents in the study were aged between 14–19 years and showed that these students are actively engaged in education. The educational status of parents of most of the students was low, falling between illiterate and elementary school. In agreement with this study, other researchers reported low educational levels among parents in northwestern Ethiopia (32). This low level of education may lead to increased khat chewing in youths because of their low level of knowledge on the effects and causes of khat chewing.

Poor academic performance of khat chewers was 20.7%, which was lower than that of the Hawassa University students (55%) and Kingdom of Saudi Arabia’s Jazan area (39.4%) (10) and higher than that of medical students (5.2%) in studies conducted in Gondar town (5.2%) (33).

Poor academic performance of non-chewers was 33.3%. Approximately 79.3% of khat-chewer students scored better in academic performance than those in the study in Hawassa, who scored 45%. However, the better academic performance of non-chewers was 90.6%, which is higher than that of the study in Hawassa 66.7% (18). Female sex was significantly associated (p <0.05) with academic performance among both khat chewers and non-chewers. The finding of this study showed that the chance of having excellent academic performance for female students was 48% [AOR=0.52:95%CI: -1.27–0.06] and 35% lower [AOR=0.65:95%CI: -0.819–0.055] than that of male students. This result is similar to the results found in the Sidama zone in southern Ethiopia (34).

About 6.2% of non-khat chewer students perform well academically, whereas only 4.1% of khat users scored well academically, which is 2.1% lower than non-users. Previous studies have also reported similar findings that khat users have poor academic performance (32). Khat-chewing students in our study have a poor chance of performing well if their academics scoring is below 50%. Most studies conducted in Ethiopia and other countries reported that students use khat for its stimulant effect, to wake up or to avoid napping, to become energized for reading, and to increase their academic performance (32). There was a statistically significant association (P<0.05) between paternal educational level and academic performance among non-khat chewers. Students whose fathers had an educational level above Grade 7 had an increased chance of achieving well academically. This result is also in line with previous studies by Hall (2022), who stated that parents who have received formal education are aware of their expectations for their adolescents’ education and strive to improve their academic success. A good educational status of parents increases that of their offspring (35).

Psychological and substance-related effects (e.g., alcohol use, depressive, and anxiety disorders) of students were statistically associated (P<0.05) with academic performance. This finding is consistent with those of previous studies (12, 32, 36). Furthermore, this study reported that the 2001–5000 ETB parental monthly income status was also statistically associated (P<0.05) with excellent academic performance, similar to studies conducted at Iowa State University in the United States. Economic constraints seriously affect students’ academic performance, ranging from the limitation of purchasing necessary educational materials to those affecting their health. Students from poor families failed to have necessities such as educational materials.

The study also revealed that school environment was statistically associated with students’ academic performance (P < 0.05). Students who were not satisfied with the school environment were 64% less likely to achieve an excellent academic performance than those who were satisfied with the school environment. According to a study by the Federal Minister of Education (2015), the school environment describes the general environment of an institution, and children were motivated to perform better in class in a positive school environment (37).

The results of this study suggest that a poor academic performance among non-khat and khat chewers with anxiety and depression was possibly because adolescents who display symptoms of depression and anxiety are predisposed to cognitive distortions and poor attention, concentration, and unending worries that undermine academic performance (38). These two overlapping symptoms of psychiatric disorders may result in various academic issues that may affect children’s academic performance. Researchers have discovered that symptoms of anxiety and depression, which can result in poor attendance, lack of motivation and interest, headaches, and fatigue, affect students’ academic performance in schools, colleges, and universities. Thus, students’ academic performance will be impacted by these circumstances (39, 40).

The results of this study also revealed poor academic performance among non-khat and khat chewers with alcohol use disorders; however, whether the links between alcohol use and academic achievement are causal relationships remain unclear. It could be that a straightforward cause–effect relationship exists or such links might be moderated by sleep, given that alcohol use is related to sleep habits, and such habits and resultant daytime sleepiness could mediate alcohol’s possible influence on academic performance (41). In addition, heavy alcohol use was associated with lower Grade point average (GPA), both directly and indirectly, through its association with fewer study hours.

Bidirectional relationships may also exist, where alcohol use and academic performance impairments mutually influence each other in a vicious circle. However, the level of academic performance in high school children predicts their drinking problems as young adults independent of individual/family confounders (42). On the other hand, bidirectional effects are supported by a longitudinal study showing that missing classes or falling behind in schoolwork were a result of drinking during college; however, the same study also showed that high school academic performance was negatively associated with heavy alcohol use (41). In general, this study found that students who chew khat experience significant clinical and social implications for enhancing their academic performance. The results suggest that professionals who provide services to patients with severe mental illness should incorporate students’ psychosocial support. Health managers and policymakers are also expected to consider this issue in their plans on developing strategies for community support programs to increase and enhance social relationships and improve public awareness and education to prevent khat use among students.

### Limitations of the study

The cross-sectional nature of the study limits the determination of the true cause–effect relationship between the dependent and independent variables. The study also has a social desirability bias to disclose information that is perceived as a socially/religiously sensitive issue and is also prone to recall bias.

### Conclusions and recommendations

This study revealed that the magnitude of poor academic performance was higher among khat chewers than among non-chewers. We found that sex, anxiety, depression, alcohol use disorder, khat chewing, and poor school environment were risk factors for poor academic performance for khat chewers.

In addition, sex, father’s educational status, monthly family income, depression, alcohol use disorder, and school environment were identified as factors associated with academic performance among non-chewers. Fathers who had an educational level of Grade 7 had a lower ability to provide motivation for an excellent academic performance for their children.

Both parental income and school environment play crucial roles in shaping students’ academic performance. While a higher parental income can provide external resources and stability, a positive school environment can offer the internal support and motivation for students to succeed. Addressing both these factors can help create a more equitable and effective educational system.

Psychiatric mental health professionals and other concerned bodies should consider creating community awareness for the prevention and treatment of khat chewing and mental illnesses.

The Amhara Health Bureau should consider strategies and policies to enhance community awareness, prevention, and control of khat chewing.

The Motta administrative education office, school administrators, and teachers must develop and teach the community how to support their children’s teaching–learning process.

The regional education bureau should design policies that prevent the availability of khat around school compounds and assign guidance to schools for risky chat chewers. Students who feel supported by their teachers are more likely to engage in learning and perform well academically. Therefore, teachers should be supportive.

A school environment that is safe, inclusive, and free from bullying encourages students to attend school regularly and participate actively, so school administrators should create a safe school environment. The Ministry of Education should create adequate facilities because schools with good infrastructure, such as libraries, laboratories, and sports facilities, provide students with the tools they need to excel. Further studies should be conducted using a mixed-study design to explore other socio-cultural aspects and religious perspectives of khat chewing that cannot be addressed by a quantitative study.

## Data Availability

The raw data supporting the conclusions of this article will be made available by the authors, without undue reservation.
